# Computational study of hybrid DDQ/LiI/WO₃ composites for multi-radiation shielding in microelectronic applications

**DOI:** 10.1038/s41598-026-68436-3

**Published:** 2026-09-08

**Authors:** K. R. M. Abdelgawad, G. Lattery, A. A. El-Daly

**Affiliations:** 1 Physics Department, Faculty of Science, Zagazig University, Zagazig, Egypt; 2 Radiation Safety Department, Research Protections, University of Pittsburgh, Pittsburgh, USA

**Keywords:** Radiation shielding, Electronics protection, Hybrid organic-inorganic composites, Charged particle attenuation, PHY/X-SRIM-MCNP simulation, Engineering, Materials science, Physics

## Abstract

Effective radiation shielding is essential for ensuring the long-term reliability and operational safety of sensitive electronic systems operating in radiation-harsh environments, including radiotherapy facilities, nuclear reactors, and space missions. In this study, novel DDQ/LiI/WO₃ hybrid composites containing 0–35 wt% WO₃ were proposed and systematically evaluated as lightweight multi-radiation shielding materials against photons, charged particles, and neutrons. Photon attenuation was investigated using MCNP and PHY-X, while SRIM and ESTAR were used to assess charged-particle interactions, and the fast-neutron removal cross section was evaluated for neutron attenuation. Increasing WO₃ loading significantly enhanced photon attenuation; at 0.1 MeV, the optimized MC-W35 composite achieved a mass attenuation coefficient of 1.778 cm²/g and a low half-value layer of 0.135 cm. From a mass-efficiency perspective, MC-W35 exhibited a notably low areal density of 0.39 g/cm² per HVL, corresponding to more than 90% lower shielding mass than conventional epoxy and polyimide matrices and approximately a ten-fold reduction relative to ordinary concrete. Secondary-radiation analysis further showed that photon-induced radiation yield decreases rapidly above 1 MeV, while electron-induced Bremsstrahlung remains suppressed, reaching less than 0.16 at 10 MeV, owing to the low average atomic number of the DDQ matrix. The enhanced fast-neutron attenuation and reduced charged-particle ranges further demonstrate the multi-radiation shielding capability of the composite. Overall, these findings identify DDQ/LiI/WO₃ hybrid composites, particularly MC-W35, as promising lightweight candidates for radiation protection of electronic systems and components operating in diverse medical, industrial, nuclear, and space radiation environments.

**Supplementary Information:**

The online version contains supplementary material available at https://doi.org/10.1038/s41598-026-68436-3.

## Introduction

Radiation plays an important role in modern life, with applications spanning medical diagnosis and radiotherapy, electricity generation in nuclear reactors, industrial inspection, sterilization, and scientific research^1–3^ However, exposure to ionizing radiation can adversely affect both human health and electronic systems. In humans, ionizing radiation can damage cellular DNA and increase the risk of tissue injury and long-term health effects. Similarly, radiation can induce ionization, charge buildup, displacement damage, and transient or permanent failures in electronic components, compromising their reliability and operational lifetime^4–6^.

Therefore, the increasing and essential use of radiation in modern life has driven the development of effective shielding approaches to protect both humans and radiation-sensitive electronic systems. Conventional radiation-shielding materials include glasses, metallic alloys, nanocomposites, ferrites, and single crystals, each offering specific advantages and limitations. High-Z glasses provide effective photon attenuation and allow composition tailoring, but their relatively high density and brittleness can restrict their use where lightweight protection is required^7–10^. Metallic alloys can provide high attenuation together with useful mechanical integrity, although their density and weight may limit their suitability for weight-sensitive applications^10–13^. Nanocomposites and ferrite-based materials offer greater compositional flexibility and the potential for multifunctional performance, although their shielding efficiency can depend strongly on filler content, dispersion, and microstructure^14,15^. Single crystals provide well-defined radiation-interaction characteristics, particularly for neutron transport, but their specialized fabrication and limited shape flexibility can restrict broader practical implementation^16,17^.

An alternative strategy to conventional external shielding is the concept of self-shielding, in which the electronic component or its surrounding material is designed to possess intrinsic radiation-protection capability. Kim^18^, proposed radiation-tolerant electronic components based on materials such as silicon carbide, while Wang and Xiao^19^ reported high radiation tolerance in carbon-nanotube-based transistors^19^. Such approaches are particularly attractive because they can reduce the need for bulky external shielding and integrate radiation protection directly into the electronic architecture. However, for a material to function effectively as a self-shielding component or encapsulating layer, high radiation attenuation alone is insufficient. It should also provide adequate mechanical integrity, thermal stability, electrical compatibility, and structural homogeneity to ensure reliable protection without compromising the performance and lifetime of the electronic device. This multifunctional requirement remains challenging for conventional shielding systems and emphasizes the necessity of hybrid materials in which radiation-attenuating constituents are integrated within a functional matrix, providing a possible material strategy for compact, lightweight, and multifunctional self-shielded electronic systems.

To address this gap, the present study proposes a hybrid DDQ/LiI/WO₃ composite as a candidate multifunctional radiation-shielding material for radiation-tolerant electronic systems. DDQ provides semiconducting behavior, charge-transport capability, thermoelectric activity, and thermal stability, together with low density and flexibility, making it attractive as a functional matrix for lightweight electronic applications^20,21^; b), LiI contributes favorable neutron-interaction characteristics^22^, whereas WO₃ enhances photon attenuation because of its high atomic number and density^23–25^. The main contribution of this study lies in coupling a charge-transfer organic matrix with high-$\:\mathrm{Z}$ fillers and neutron absorbers to achieve balanced multi-radiation attenuation (photons, neutrons, charged particles) and secondary Bremsstrahlung suppression within a single, ultra-lightweight architecture. Radiation-shielding materials can be investigated through complementary experimental, theoretical, and simulation-based approaches. Experimental measurements provide direct evaluation of attenuation performance under controlled irradiation conditions, while theoretical calculations allow shielding parameters to be predicted from material composition and related physical properties^26,27^. Radiation-transport simulations, particularly Monte Carlo methods, enable detailed analysis of particle interactions and shielding performance over broad energy ranges before practical fabrication^28^. In the present study, the proposed composite was investigated using computational and simulation-based approaches, employing Photon Shielding and Dosimetry software (Phy-X/PSD), the Monte Carlo N-Particle Transport Code (MCNP), the Stopping and Range of Ions in Matter (SRIM) program, and the Electron Stopping Powers and Ranges (ESTAR) database over a broad energy range covering photons, fast neutrons, protons, alpha particles, and beta particles. This physics-based assessment provides a comprehensive evaluation of the proposed composite and establishes a foundation for subsequent experimental fabrication and validation of lightweight, multifunctional shielding materials for electronic systems operating in radiation-harsh environments.

## Materials and methods

This study employs a comprehensive theoretical framework to evaluate the radiation shielding efficacy of the proposed DDQ-LiI-WO₃ composite materials. The methodology consists of two main parts. First, the design and characterization of theoretical samples based on their elemental compositions. Second, a multi-modal analysis of their attenuation properties against gamma rays, fast neutrons, and charged particles, utilizing a combination of established databases and Monte Carlo simulations.

### Compositional modeling and sample design

Five theoretical DDQ–LiI–WO₃ composite systems were designed and modeled to evaluate their potential as radiation shielding materials for radiation-exposed microelectronic applications. These composites are designated as MC-W00, MC-W05, MC-W15, MC-W25, and MC-W35. The general notation MC-WX is used, where “MC” denotes Microelectronics Composite and “WX” represents the weight fraction of tungsten trioxide (WO₃) incorporated into the composite (e.g., MC-W35 contains 35 wt% WO₃). Each formulation consists of 2,3-dichloro-5,6-dicyano-1,4-benzoquinone (DDQ), lithium iodide (LiI), and WO₃, with detailed compositions provided in Table 1. A comprehensive theoretical modeling approach was developed to describe a synergistic, multi-modal radiation shielding response achieved through the integration of materials with complementary shielding functions. The selection of DDQ, LiI, and WO₃ was strategically guided by their combined ability to address multiple radiation interaction mechanisms. The DDQ organic matrix functions as a lightweight host material, facilitates uniform dispersion of inorganic fillers, and promotes efficient charge dissipation at the molecular level. Lithium iodide primarily enhances neutron interaction efficiency, while tungsten trioxide contributes significant attenuation of photons and charged particles. Optimization of filler dispersion and matrix–filler compatibility is critical for preserving the mechanical robustness and thermal stability necessary for multifunctional applications (Desoky et al., 2021;^22,25^.

**Table 1:** Chemical composition and calculated densities (g/cm^3^) of the composite samples.

Sample Code	DDQ (wt%)	LiI (wt%)	WO₃ (wt%)	Density (g/cm³)
MC-W00	0.750	0.250	0	1.990
MC-W05	0.700	0.250	0.050	2.083
MC-W15	0.600	0.250	0.150	2.298
MC-W25	0.500	0.250	0.250	2.562
MC-W35	0.400	0.250	0.350	2.895

The theoretical densities ($\:{\rho\:}_{\mathrm{theo}}$) of the composite systems were computed using the inverse form of the rule of mixtures, assuming an ideal, void-free structural matrix^29^:

1 $$\:{{\uprho\:}}_{\mathrm{theo}}=\frac{1}{\sum\:_{\mathrm{i}}\frac{{\mathrm{w}}_{\mathrm{i}}}{{{\uprho\:}}_{\mathrm{i}}}}=\frac{1}{\frac{{\mathrm{w}}_{\mathrm{DDQ}}}{{{\uprho\:}}_{\mathrm{DDQ}}}+\frac{{\mathrm{w}}_{\mathrm{LiI}}}{{{\uprho\:}}_{\mathrm{LiI}}}+\frac{{\mathrm{w}}_{{\mathrm{WO}}_{3}}}{{{\uprho\:}}_{{\mathrm{WO}}_{3}}}}\:$$

where $\:{\mathrm{W}}_{\mathrm{i}}$ is the weight fraction ($\:\sum\:{\mathrm{W}}_{\mathrm{i}}=1$) and $\:{{\uprho\:}}_{\mathrm{i}}$ is the density of the i^th^ component, respectively. The individual component densities utilized for the formulation design were $\:{{\uprho\:}}_{\mathrm{DDQ}}=1.7\mathrm{\:g}\cdot\:{\mathrm{cm}}^{-3}$ for the organic matrix, $\:{{\uprho\:}}_{\mathrm{LiI}}=4.08\mathrm{\:g}\cdot\:{\mathrm{cm}}^{-3}$ for lithium iodide, and $\:{{\uprho\:}}_{{\mathrm{WO}}_{3}}=7.16\text{}\mathrm{g}\cdot\:{\mathrm{cm}}^{-3}$ for tungsten trioxide. The calculated bulk densities of the resulting composite formulations (MC-W00 to MC-W35) are summarized in Table 1.

### Density and compositional effects

Table 1 shows the nominal compositions and the theoretically calculated densities of the DDQ-LiI-WO₃ composite systems. The results indicate a well-defined dependence of composite density on the tungsten trioxide (WO₃) content, with density increasing progressively as the WO₃ weight fraction rises. This behavior is primarily governed by the physical attributes of tungsten, including its high atomic number (Z = 74) and relatively high density (7.16 g·cm⁻³). The observed density evolution serves as a critical validation of composite design methodology, demonstrating compliance with fundamental radiation physics considerations. Hence, it is expected that increasing the density could enhance the probability of interaction between incident radiation and atomic nuclei within the material, thereby providing a foundational mechanism for the expected improvement in radiation attenuation performance.

### Gamma-ray attenuation analysis

A dual-method theoretical approach was employed to evaluate the radiation shielding effectiveness of the proposed composite materials, combining estimations from established databases with Monte Carlo simulations. PHY-X/PSD software was utilized to calculate the main attenuation parameters for a wide range of energy, from 0.01 to 10 MeV. These parameters are the mass attenuation coefficient (µ_m_), the linear attenuation coefficient (µ), the half-value layer (HVL), the mean free path (MFP), and the effective atomic number (Z_eff_). These programs use the XCOM database and provide reliable information about materials with more than one element.

#### Simulation setup and computational methodology

Monte Carlo simulations were performed using the MCNP5 code to evaluate photon transmission and calculate the linear attenuation coefficient () of the proposed DDQ–LiI–WO composites. The simulation geometry was designed to represent a narrow-beam transmission arrangement.

A monoenergetic collimated photon source was positioned at cm and directed along the positive -axis. Photon energies ranging from 0.01381 to 10 MeV were simulated sequentially. The shielding sample was modeled as a rectangular block with dimensions of 6 × 6 × 1 cm³, and the elemental composition and theoretically calculated density of each composite were assigned in the material definition card. The computational model was enclosed within a 100 × 100 × 100 cm³ air-filled domain surrounded by an outer void region to eliminate boundary effects. A detector surface was positioned 2 cm behind the sample to record the transmitted photon current.

Photon transport was simulated using MODE P, and the particle importance (IMP: P) variance-reduction technique was employed to improve computational efficiency. The transmitted photon current was scored using the F1:P surface current tally. Each simulation was performed with particle histories, providing high statistical precision. The statistical uncertainty, expressed as the Fractional Standard Deviation (FSD), remained below 0.6% for energies of (0.1–10 MeV), while higher uncertainties * 0.126* ($\:12.6\mathrm{\%}$) occurred at selected lower energies, ($\:\mathrm{E}<0.1\mathrm{\:MeV}$), because of low transmitted-photon counts.

#### Radiation shielding quantities and attenuation analysis

The linear attenuation coefficient was calculated according to the Beer–Lambert law^30^:

2 $$\:{\upmu\:}=-\frac{1}{\mathrm{d}}\:\mathrm{ln}\left(\frac{\mathrm{I}}{{\mathrm{I}}_{\mathrm{O}}}\right)$$

where d is the sample thickness, I and I_O_ denote the incident and transmitted photon intensities obtained from the MCNP F1:P surface current tally, respectively.

The mass attenuation coefficient (µₘ, cm²/g) characterizes the probability of photon interactions within a material and serves as a key parameter in radiation–matter interaction studies^31^. It is defined as

3 $$\:{{\upmu\:}}_{\mathrm{m}}=\frac{{\upmu\:}}{{\uprho\:}}\:$$

Where µ is the linear attenuation coefficient (cm^**− 1**^), and ρ is the density (g/cm³). Materials having higher µ_m_ values are better at stopping photons. The half-value layer (HVL), or the thickness needed to reduce the photon beam intensity by 50%, was calculated as

4 $$\:\mathrm{H}\mathrm{V}\mathrm{L}=\frac{0.693}{{\upmu\:}}$$

The mean free path (MFP) represents the average distance traversed by a photon before interacting with the material^32^ and is given by:

5 $$\:\mathrm{M}\mathrm{F}\mathrm{P}\:=\frac{1}{{\upmu\:}}$$

The effective atomic number (Z_eff_) of each composite was estimated using the ratio of atomic and electronic cross-sections:

6 $$\:{\mathrm{Z}}_{\mathrm{e}\mathrm{f}\mathrm{f}}=\frac{\:{{\upsigma\:}}_{\left(\mathrm{t},\:\mathrm{a}\right)}}{{{\upsigma\:}}_{\left(\mathrm{t},\mathrm{e}\mathrm{l}\right)}}$$

Z_eff_varies with energy and composition, offering a comprehensive indicator of photon interaction probabilities (Abdelgawad et al.^33^).

### Neutron attenuation analysis

Fast neutron attenuation in the proposed composites was modeled considering both scattering and absorption processes. The optimal shielding performance is achieved by the incorporation of hydrogen-rich moderators, which slow down fast neutrons well, along with elements that absorb neutrons. The effectiveness of a material in eliminating fast neutrons is characterized by the removal cross-section (Σ_R_), which represents the likelihood that an incident neutron is removed from the primary beam after its initial impact. The removal cross-section values for the studied composites were determined using a formula proposed by^34^:

7 $$\:{{\sum}}_{\mathrm{R}}={\sum\:}_{\mathrm{i}}{\:{\uprho\:}}_{\mathrm{i}}\:{\left(\frac{{{\sum}}_{\mathrm{R}}}{{\uprho\:}}\right)}_{\mathrm{i}}$$

In this equation, $\:{{\uprho\:}}_{\mathrm{i}}$ represents the partial density of the i^th^ constituent in the composite, calculated as $\:{{\uprho\:}}_{\mathrm{i}}={\mathrm{w}}_{\mathrm{i}}\:{{\uprho\:}}_{\mathrm{c}\mathrm{o}\mathrm{m}\mathrm{p}\mathrm{o}\mathrm{s}\mathrm{i}\mathrm{t}\mathrm{e}}.$ The elemental mass removal cross sections, (Σ_R_/ρ)_i_, for the constituent elements (Cl, C, O, Li, N, I, and W) were calculated using the empirical mathematical relations established by El Abd et al., By applying these specific elemental parameters, the effective removal cross-section (Σ_R_) for each composite was subsequently derived^34–36^; El Abd, 2016).

### Charged particle attenuation analysis

The shielding capacity for charged particles (alpha particles, protons, and beta particles) was evaluated using mass stopping power, which is defined as the energy loss per unit distance.

For heavy charged particles (protons and alpha particles), the analysis relies on the standard Bethe–Bloch equation (Bethe’s formula), which models the collisional energy loss. Due to the complexity of the full equation and the use of specialized software, the analysis was conducted using the SRIM code, which applies the full Bethe–Bloch formulation, including necessary corrections like the shell correction and density effect, to calculate the mass stopping power (MSP).

For electron (β-particle) interactions, the ESTAR program developed by the NIST was employed to calculate the total mass stopping power. This approach accounts for both collisional energy losses, described by the Bethe equation with appropriate corrections, and radiative losses arising from Bremsstrahlung processes. The inclusion of Bremsstrahlung effects is essential for accurately modeling the energy loss of light-charged particles such as electrons, even at intermediate energies.

The fundamental form of the energy loss per unit distance (stopping power) for charged particles is given:

8 $$\:-\frac{\mathrm{d}\mathrm{E}}{\mathrm{d}\mathrm{x}}=4{\uppi\:}{\mathrm{r}}_{\mathrm{e}}^{2}{\mathrm{m}}_{\mathrm{e}}{\mathrm{c}}^{2}\frac{{\mathrm{Z}}^{2}{\mathrm{N}}_{\mathrm{A}}{\mathrm{Z}}_{\mathrm{mat}}}{{\mathrm{A}}_{\mathrm{mat}}{\hspace{0.17em}}{{\upbeta\:}}^{2}}\left[\mathrm{l}\mathrm{n}\left(\frac{2{\mathrm{m}}_{\mathrm{e}}{\mathrm{c}}^{2}{{\upbeta\:}}^{2}{{\upgamma\:}}^{2}}{\mathrm{I}}\right)-{{\upbeta\:}}^{2}-2\frac{{\mathrm{Z}}_{\mathrm{m}\mathrm{at}}}{{\mathrm{A}}_{\mathrm{mat}}}\mathrm{C}-{\updelta\:}\right]\:$$

Where Z is the projectile’s charge number, β is the projectile’s velocity relative to the speed of light, I is the mean excitation energy of the target material, r_e_ is the classical electron radius, m_e_c^2^ is the electron rest energy, N_A_ is Avogadro’s number, Z_mat_ and A_mat_ are the atomic number and mass number of the material, C is the shell correction term, and **δ** is the density effect correction term^37^.

The range of a particle, defined as the total distance that travels before stopping, is calculated by:

9 $$\:\mathrm{R}\left(\mathrm{T}\right)={\int\:}_{0}^{\mathrm{T}}{\left(-\frac{\mathrm{d}\mathrm{E}}{\mathrm{d}\mathrm{x}}\right)}^{-1}\:\mathrm{d}\mathrm{E}$$

Where $\:\mathrm{T}$ denotes the initial kinetic energy of the incident charged particle, and $\:-\mathrm{d}\mathrm{E}/\mathrm{d}\mathrm{x}$ is its stopping power in the shielding material. In practice, SRIM performs this integration numerically to estimate the projected range and energy loss of ions within the target material. Scaling relations were applied to compare the ranges and stopping powers of particles with different masses and charges, linking the theoretical predictions from the Bethe–Bloch model with SRIM and ESTAR simulation outcomes^38^; Abdelgawad et al.^39^,.

## Results and discussion

### Gamma-ray attenuation

**Fig. 1:**
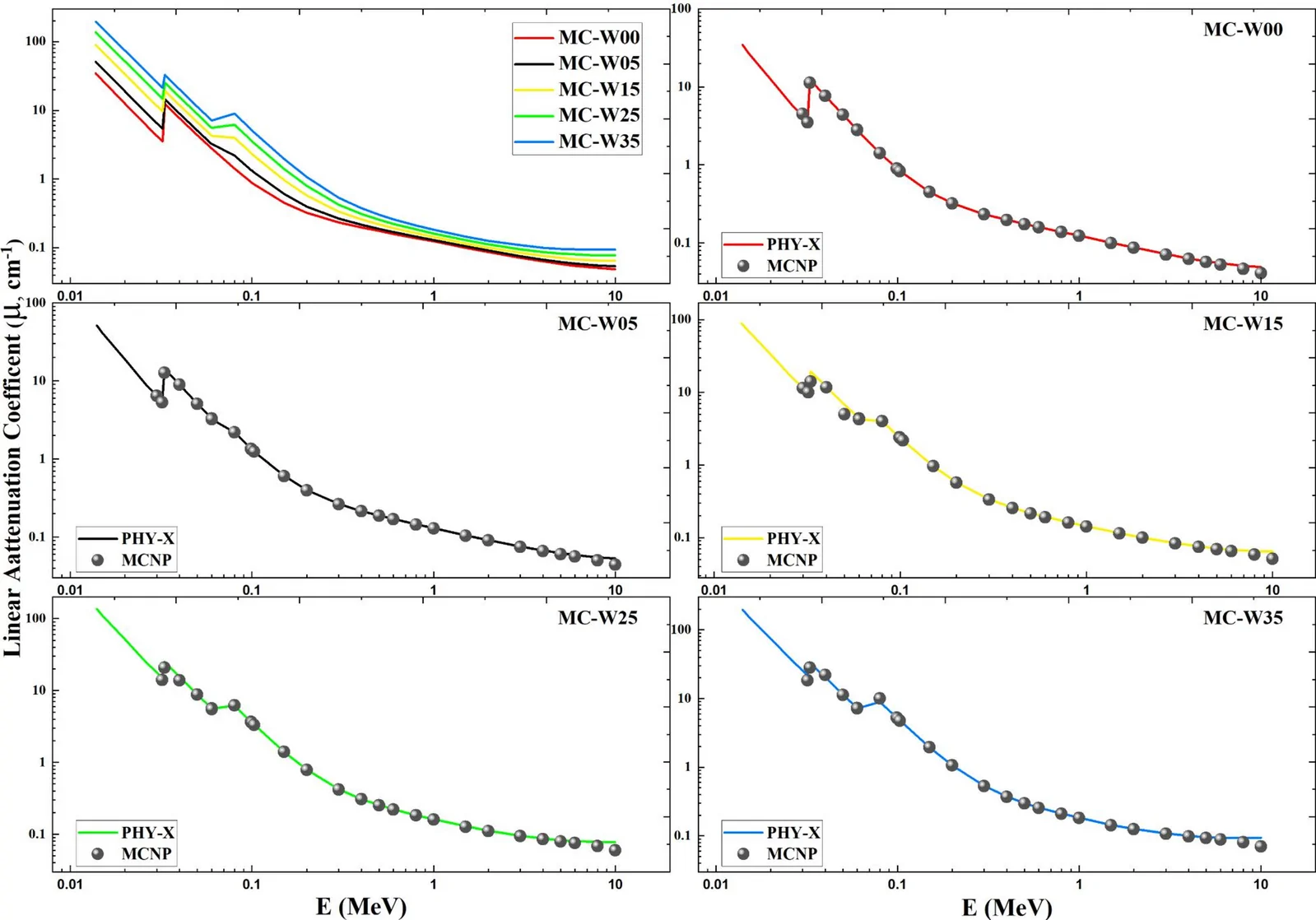
linear attenuation coefficient (µ, cm^−1^) as a function of photon energy (MeV) for MC-W composites. The solid curves represent theoretical values obtained via PHY-X/PSD, while the symbols represent the MCNP simulation data.

The variation of the linear attenuation coefficient (µ) with photon energy (0.014–10 MeV) reveals three distinct interaction regimes governed by photon–matter interaction mechanisms (Fig. 1). In the low-energy region (0.014–0.1 MeV), photoelectric absorption dominates and increases strongly with atomic number while decreasing with photon energy (σₚₑ ∝ Z⁴–⁵/E³·⁵). Therefore, the incorporation of high-Z WO₃ (ZW = 74) markedly enhances photon attenuation. MC-W35 shows a substantially higher µ than the WO₃-free matrix (MC-W00) at the lowest energies. The sharp discontinuities in the attenuation profiles correspond to the K-absorption edges of iodine (33.2 keV) and tungsten (69.5 keV), where the photon energy approaches the binding energy of inner-shell electrons^40^. In the intermediate region (0.1–1 MeV), µ decreases rapidly as Compton scattering becomes dominant. Since Compton scattering depends mainly on electron density rather than atomic number, the effect of WO₃ loading becomes less pronounced. However, the higher-density composites retain higher attenuation coefficients. At high energies (1–10 MeV), pair production becomes increasingly important above its 1.022 MeV threshold. Across the entire spectrum, MCNP simulations show good agreement with PHY-X/PSD database calculations. The numerical precision of the MCNP transport simulations was verified via energy-dependent relative error analysis. At lower energies ($\:\mathrm{E}<0.1\mathrm{\:MeV}$), localized statistical uncertainties peaked at $\:12.6\mathrm{\%}$ as intense photoelectric interactions near absorption edges decreased the transmitted photon current (tally histories). Conversely, for the broader energy range of $\:0.1-10\mathrm{\:MeV}$, the FSD remained strictly below * 0.006* ($\:<0.6\mathrm{\%}$), rendering the calculated error bars physically smaller than the graphical data points in Fig. 1.

**Fig. 2:**
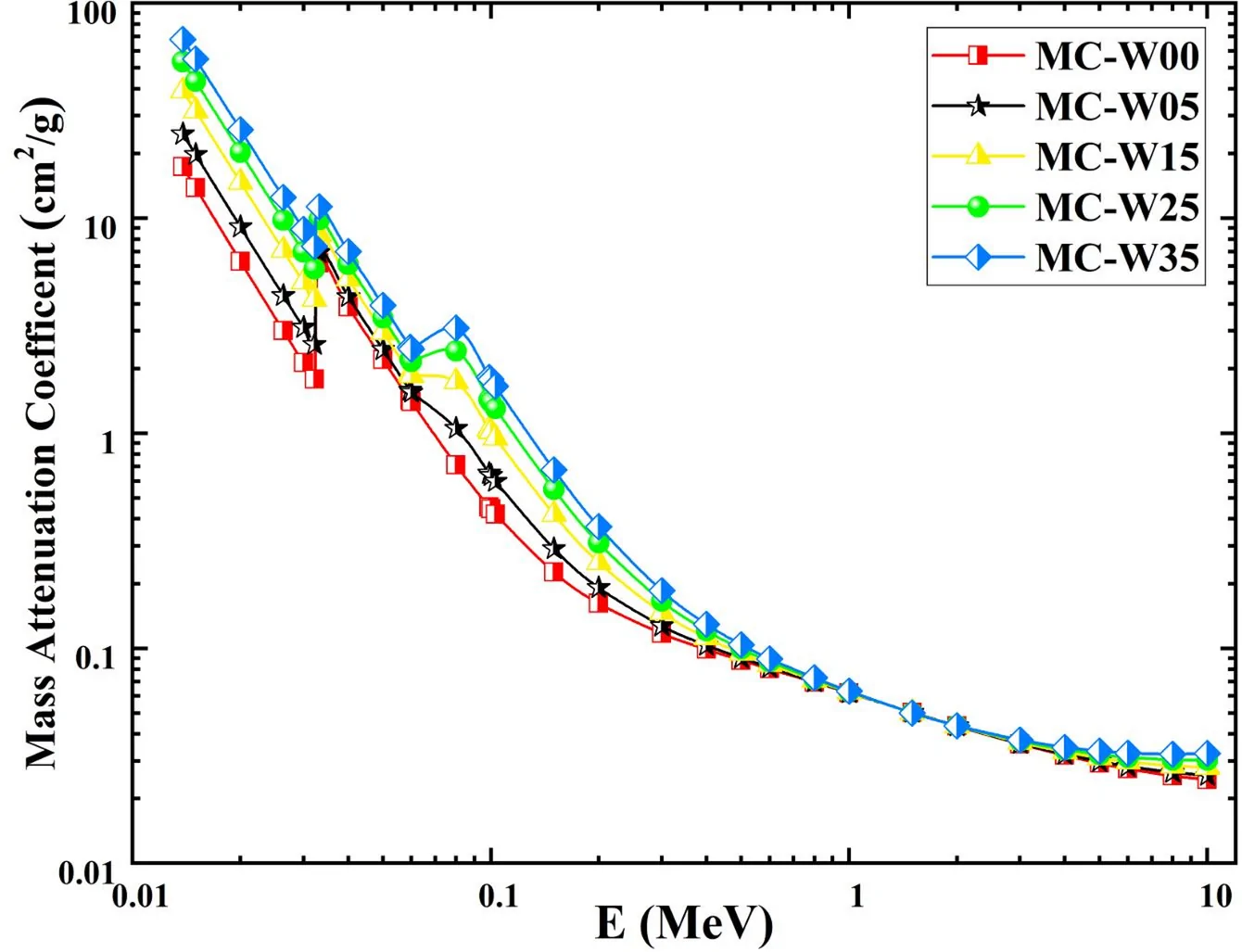
Mass attenuation coefficient (µ_m_, cm²/g) as a function of photon energy (MeV) for MC-W composites.

Figure 2 presents the mass attenuation coefficient (µₘ) of the MC-W composites across the investigated photon-energy range. Since µₘ is normalized to material density, it provides a direct measure of the intrinsic photon-interaction capability of each composition. MC-W35 maintains the highest µₘ over most of the energy range, confirming the contribution of WO₃ to photon attenuation. The characteristic K-absorption edges identified in Fig. 1 are also reflected in the µₘ profiles, consistent with the energy-dependent photoelectric interaction of the composite constituents. At higher energies, the compositional differences gradually decrease as Compton scattering becomes more influential, while pair production contributes above 1.022 MeV. Overall, increasing WO₃ content enhances the intrinsic photon-attenuation capability of the composites, particularly in the low-energy region^41^.

The MC-W35 composite was compared with materials commonly employed for microelectronic encapsulation, structural packaging, and radiation, as shown in Table 2. At 0.1 MeV, MC-W35 exhibited a mass attenuation coefficient of 1.778 cm²/g and a density of 2.89 g/cm³. Its mass attenuation coefficient was substantially higher than those of epoxy resin (0.069 cm²/g), polyimide (0.059 cm²/g), silicon (0.175 cm²/g), and ordinary concrete (0.174 cm²/g). Lead and tantalum exhibited higher mass attenuation coefficients of 5.549 and 4.3 cm²/g, respectively^44^. However, their substantially higher densities of 11.35 and 16.70 g/cm³ impose a significant weight penalty, which is an important limitation for space applications where minimizing shielding mass is critical. To further quantify mass efficiency, the areal density required per half-value layer (ρ_x, HVL_ = ρ X HVL) was evaluated. The MC-W35 composite achieved an areal density of 0.39 g/cm^2^, corresponding to a mass reduction of more than 90% compared with standard epoxy (10.04 g/cm^2^) and polyimide (11.75 g/cm^2^), and an approximately ten-fold reduction compared with ordinary concrete (4.06 g/cm^2^). Therefore, MC-W35 provides a favorable balance between photon attenuation and shielding mass, supporting its potential use where effective radiation protection and reduced shielding mass are required.

**Table 2:** Comparison of the mass attenuation coefficient ($\:{\mu\:}_{m}$), density ($\:\rho\:$), and HVL-corresponding areal density ($\:{\rho\:}_{\mathrm{areal}}$) of MC-W35 with selected reference materials at $\:0.1\mathrm{\:MeV}$.

Material	µ_m_ (cm²/g)	ρ (g/cm³)	ρ _areal_ (g/cm^2^)	Ref
Epoxy resin	0.069	1.110	10.046	El-Sawy and Sarwat^42^,
Polyimide (Kapton)	0.059	1.400	11.748	Cherkashina^43^,
Silicon	0.175	2.330	4.068	Hubbell and Seltzer^40^,
Tantalum	4.300	16.700	0.161	Hubbell and Seltzer^40^,
Lead	5.549	11.350	0.125	Hubbell and Seltzer^40^,
Ordinary concrete	0.1738	2.300	4.063	Hubbell and Seltzer^40^,
MC-W35	1.778	2.890	0.390	Current study

**Fig. 3:**
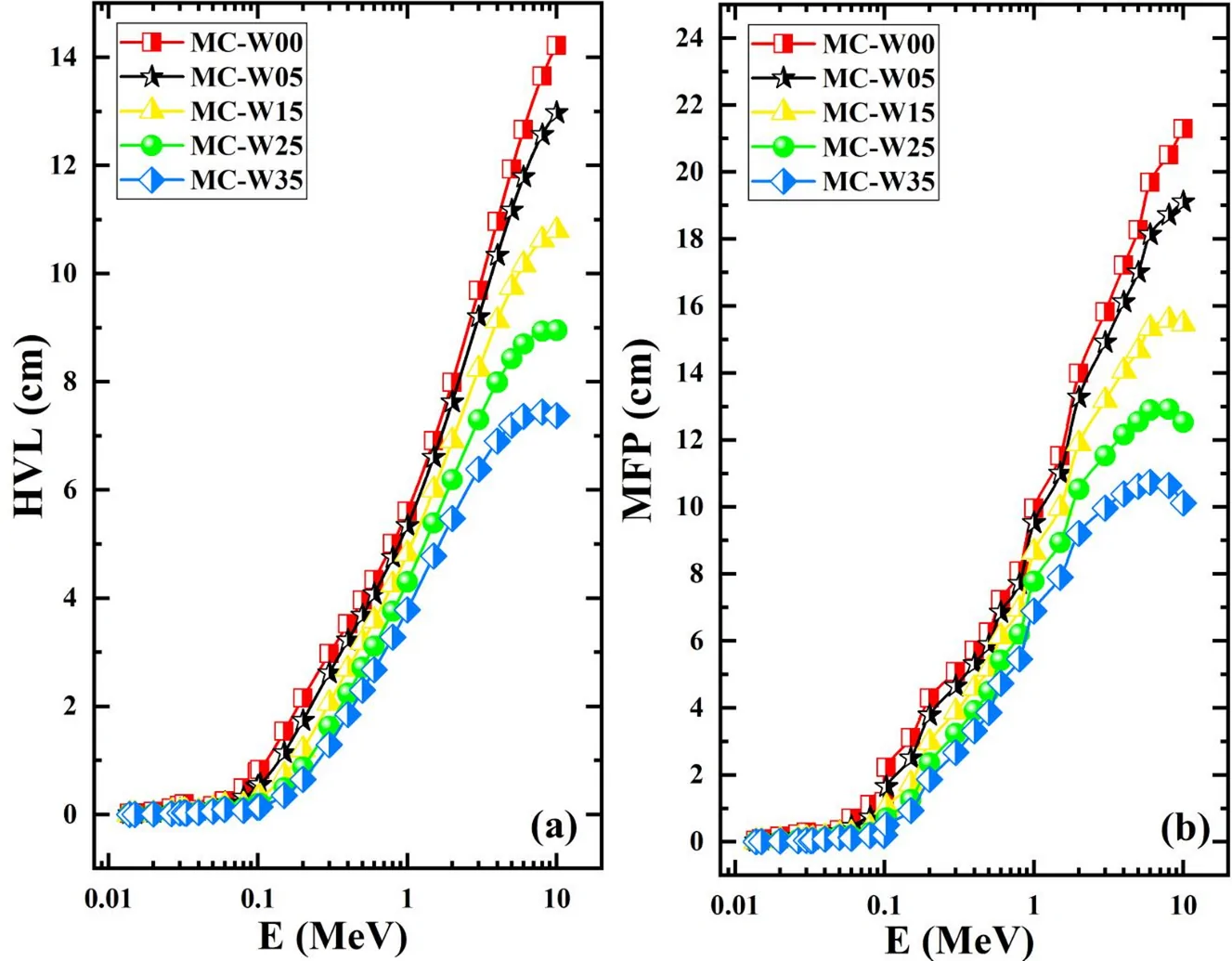
(**a**) Half-value layer (HVL, cm) and (**b**) mean free path (MFP, cm) as a function of photon energy (MeV) for MC-W composites.

Figure 3 translates the attenuation coefficients (µ and µₘ) into practical thickness parameters by presenting the Half-Value Layer (HVL, Fig. 3a) and Mean Free Path (MFP, Fig. 3b) over the investigated photon-energy range. Both parameters remain very low at low photon energies, reflecting the strong attenuation observed in Figs. 1 and 2. As photon energy increases, HVL and MFP rise markedly because the photon interaction probability decreases, requiring greater material thickness to attenuate the beam. MC-W35 consistently exhibits the lowest HVL and MFP among the investigated formulations, which reflects the higher density and stronger photon attenuation provided by its increased WO₃ content. At the highest energies, both parameters approach a gradual plateau, indicating smaller changes in attenuation with further energy increases. Overall, the reduced HVL and MFP of the WO₃-rich composites demonstrate their advantage in reducing the thickness required for effective photon attenuation^45^.

**Fig. 4:**
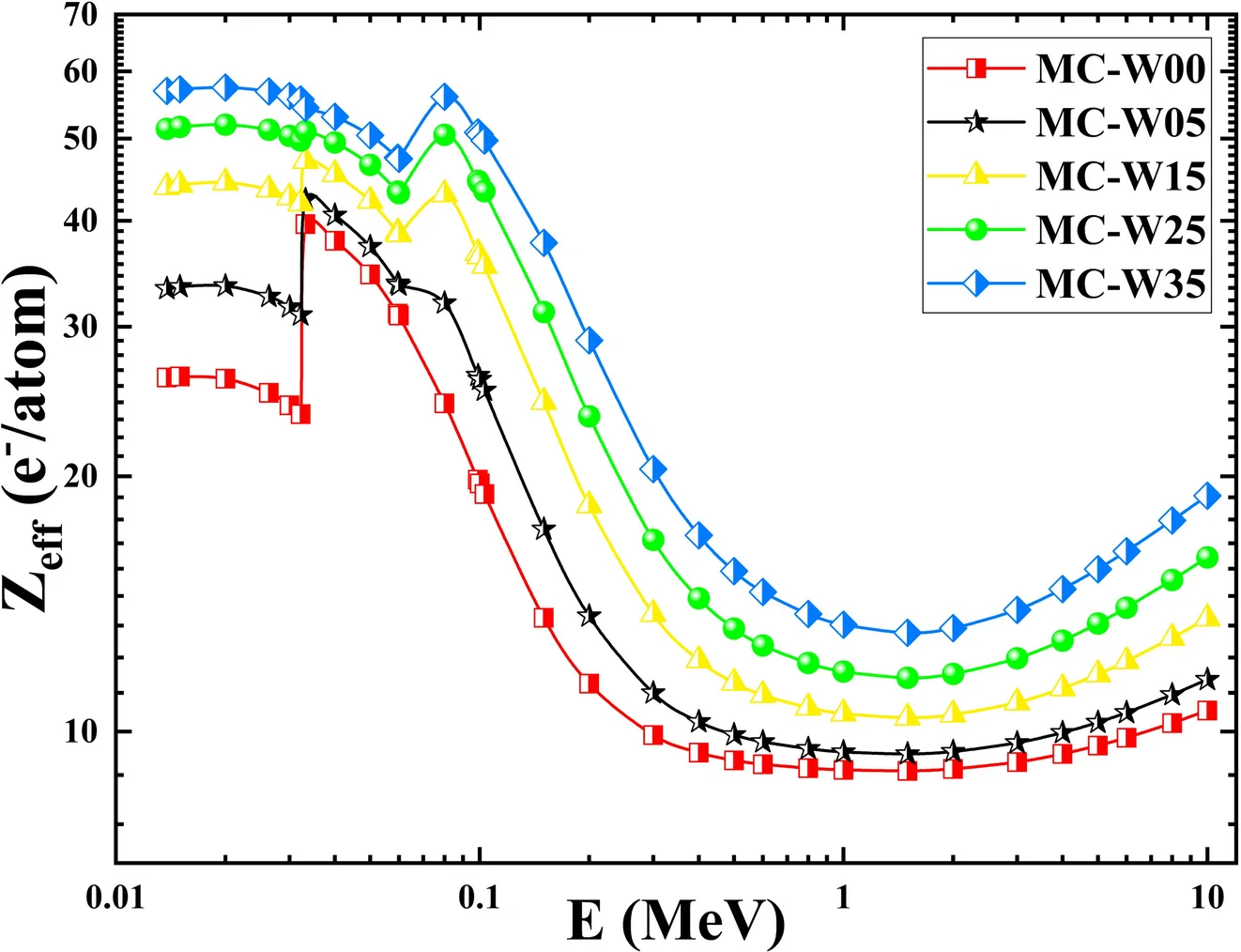
Effective atomic number (Z_eff_) as a function of photon energy (MeV) for MC-W composites.

Figure 4 illustrates the energy-dependent effective atomic number (Z_eff_) of the MC-W composites. Z_eff_ reflects the combined contribution of the constituent elements to photon interaction. The effect of WO₃ loading is clear in the low-energy region; at ~ 0.02 MeV, Z_eff_ increases from about 25 e⁻/atom for MC-W00 to nearly 57 e⁻/atom for MC-W35. This increase results from the greater contribution of high-Z W atoms to photoelectric absorption. The discontinuities near 33.2 and 69.5 keV correspond to the iodine and tungsten K-absorption edges, respectively, as identified in Figs. 1 and 2. With increasing energy, Z_eff_ decreases as Compton scattering becomes more important. For MC-W35, it decreases from about 51 at 0.1 MeV to ~ 14 at 1 MeV. At higher energies, Z_eff_ rises gradually due to the increasing contribution of pair production. At 10 MeV, it reaches ~ 18.5 e⁻/atom for MC-W35 compared with ~ 11 e⁻/atom for MC-W00. The higher Z_eff_ of WO₃-rich composites confirms their stronger composition-dependent photon interaction across the investigated energy range^46^.

### Fast neutron attenuation

Beyond photon attenuation, the fast-neutron shielding capability of the composites was evaluated using the macroscopic removal cross-section ($\:{{\Sigma\:}}_{\mathrm{R}}$), as presented in Table 3. Increasing the WO₃ content produced a modest but consistent increase in $\:{{\Sigma\:}}_{\mathrm{R}}$, from 0.0727 cm for MC-W00 to 0.0792 cm for MC-W35, corresponding to an overall improvement of approximately 9%. This behavior indicates that the incorporation of the W-containing phase enhances the probability of fast-neutron removal through additional neutron–nucleus interactions. However, the contribution of WO₃ to fast-neutron attenuation remains moderate because efficient neutron energy reduction depends strongly on the scattering and absorption characteristics of the complete composite rather than on the heavy element alone. In the present system, LiI provides an additional neutron-capture contribution, while the other constituent elements contribute to neutron scattering and energy loss. Therefore, the increase in WO₃ content improves fast-neutron removal while simultaneously providing the strong photon attenuation observed in the previous sections. This complementary behavior demonstrates the ability of the DDQ/LiI/WO₃ system to provide broader radiation protection without relying on a separate neutron-shielding component (^34^; El Abd, 2016).

**Table 3:** Procedures and calculated values used to determine the fast neutron removal cross section (Σ_R_, cm^− 1^) for the investigated samples.

Element	Σ_*R*/ρ_	MC-W00 (ρ = 1.990 g/cm^3^)			MC-W05 (ρ = 2.083 g/cm^3^)			MC-W15 (ρ = 2.298 g/cm^3^)			MC-W25 (ρ = 2.562 g/cm^3^)			MC-W35 (ρ = 2.865 g/cm^3^)		
		W_i_	ρ_i_	∑_*R* (cm−1)_	W_i_	ρ_i_	∑_*R* (cm−1)_	W_i_	ρ_i_	∑_*R* (cm−1)_	W_i_	ρ_i_	∑_*R* (cm−1)_	W_i_	ρ_i_	∑_*R* (cm−1)_
C	0.0502	0.354	0.704	0.035	0.330	0.687	0.034	0.282	0.648	0.033	0.234	0.601	0.030	0.187	0.542	0.027
Cl	0.0252	0.261	0.519	0.013	0.243	0.507	0.013	0.208	0.478	0.012	0.173	0.443	0.011	0.138	0.400	0.010
N	0.0448	0.103	0.205	0.009	0.096	0.200	0.009	0.082	0.189	0.008	0.068	0.175	0.008	0.055	0.158	0.007
O	0.0405	0.118	0.234	0.009	0.122	0.253	0.010	0.129	0.297	0.012	0.137	0.350	0.014	0.144	0.417	0.017
Li	0.0840	0.009	0.017	0.001	0.009	0.018	0.001	0.008	0.020	0.002	0.008	0.022	0.002	0.008	0.024	0.002
I	0.0133	0.156	0.310	0.004	0.156	0.324	0.004	0.155	0.357	0.005	0.155	0.397	0.005	0.154	0.447	0.006
W	0.0110				0.045	0.094	0.001	0.135	0.310	0.003	0.224	0.575	0.006	0.313	0.907	0.010
Σ_R_			**0.0727**			**0.0733**			**0.0749**			**0.0768**			**0.0792**	

**Fig. 5:**
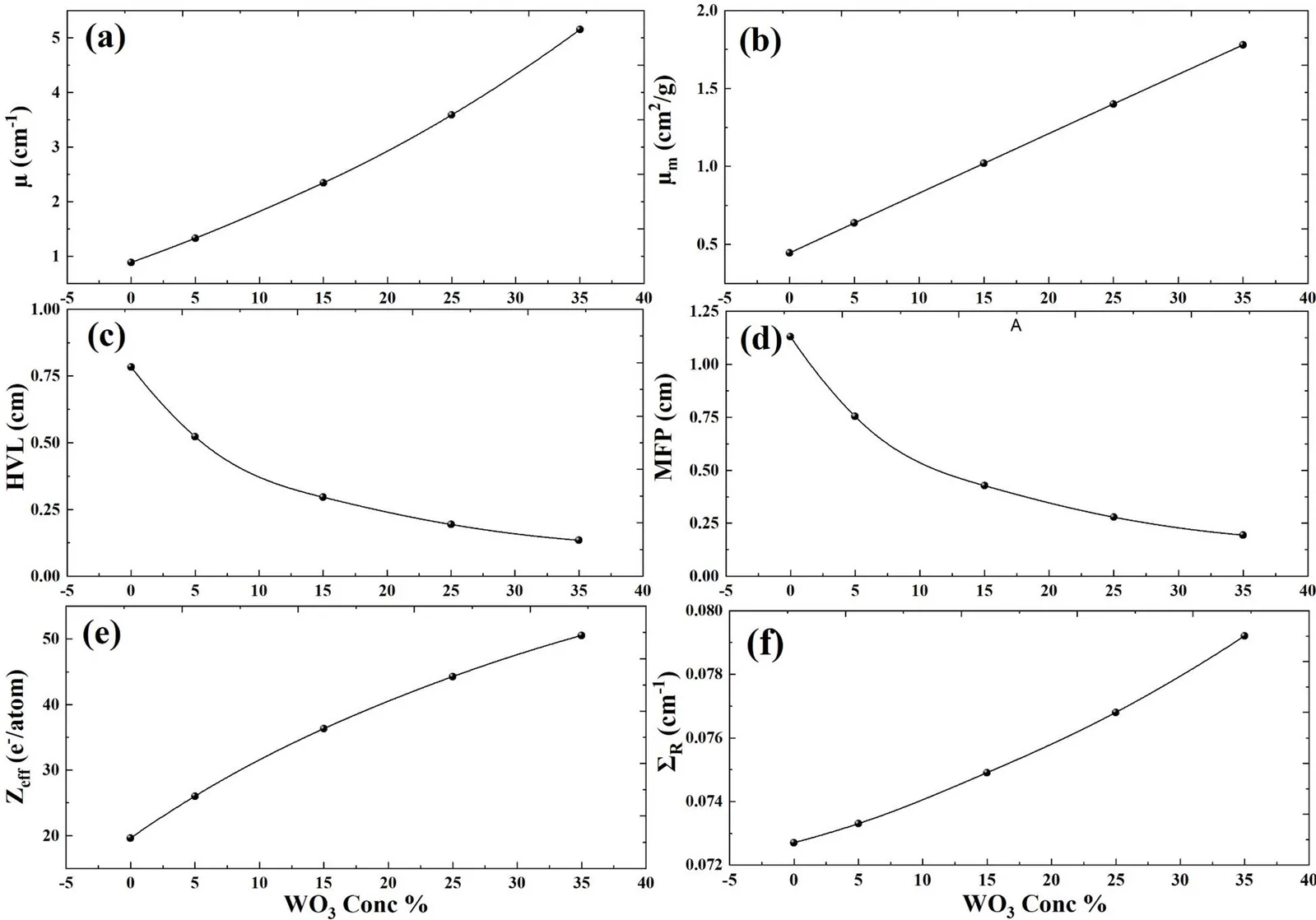
Sensitivity of key radiation shielding parameters ($\:{\upmu\:}$, $\:{{\upmu\:}}_{\mathrm{m}}$, $\:\mathrm{H}\mathrm{V}\mathrm{L}$, $\:\mathrm{M}\mathrm{F}\mathrm{P}$, $\:{\mathrm{Z}}_{\mathrm{eff}}$, and $\:{{\Sigma\:}}_{\mathrm{R}}$) to $\:{\mathrm{WO}}_{3}$ filler concentration (0–35 wt%) at 0.1 MeV photon energy.

Figure 5 presents the sensitivity of the main shielding parameters to WO₃ loading (0–35 wt%) at 0.1 MeV. At this energy, photoelectric absorption remains the dominant photon interaction mechanism, making the attenuation highly sensitive to the atomic number of the composite constituents. Increasing the WO₃ content markedly raises Z_eff_ from 19.6 to 50.4 e⁻/atom because the high-Z tungsten atoms increasingly contribute to photon absorption. This compositional effect increases the mass attenuation coefficient (µ_m_) from 0.44 to 1.78 cm²/g. The linear attenuation coefficient (µ) shows a stronger, nonlinear increase from 0.89 to 5.16 cm⁻¹ because both the intrinsic attenuation ability and the bulk density increase with WO₃ loading, according to ($\:{\upmu\:}={{\upmu\:}}_{\mathrm{m}}\cdot\:{\uprho\:}$). The resulting increase in photon interaction probability reduces the penetration depth, causing the HVL to decrease from 0.78 to 0.135 cm and the MFP from 1.12 to 0.19 cm. The fast-neutron removal cross-section (Σ_R_) also increases from 0.0727 to 0.0792 cm⁻¹, although the change is relatively small. This behavior reflects the additional inelastic scattering contribution of tungsten nuclei, while neutron moderation remains mainly associated with the lighter nuclei in the organic matrix. Overall, the sensitivity analysis confirms that increasing WO₃ loading produces a coordinated improvement in photon attenuation and a modest enhancement in fast-neutron removal.

### Charged particle attenuation

#### Alpha particles (He^+ 2^)

**Fig. 6:**
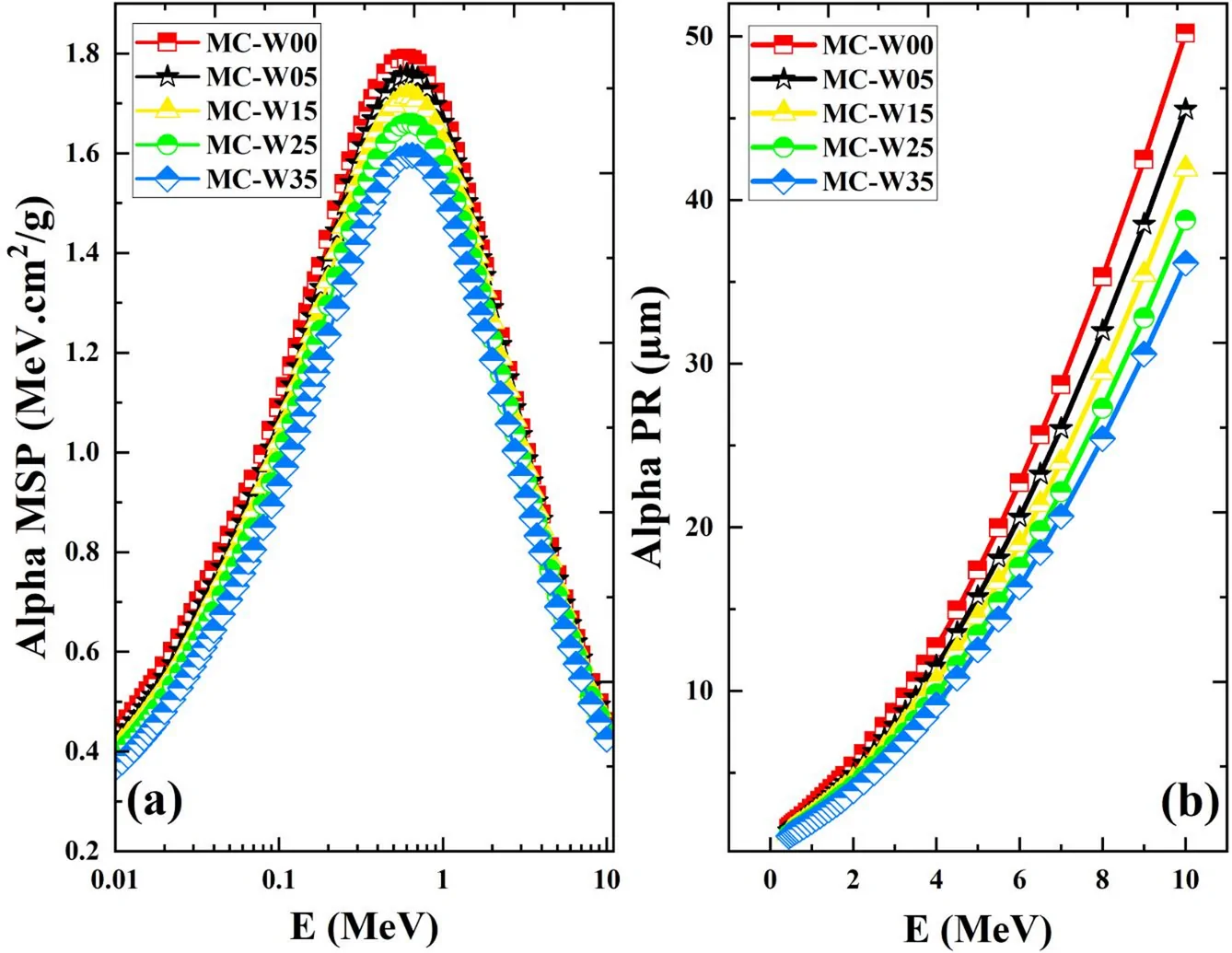
Alpha (**a**) mass stopping power (MSP, MeV·cm²/g) and (**b**) projected range (PR, µm) as a function of particle energy (MeV) for MC-W00–MC-W35 samples.

Figure 6 illustrates the mass stopping power ($\:\mathrm{MSP}$, Fig. 6a) and projected range ($\:\mathrm{P}\mathrm{R}$, Fig. 6b) of alpha particles across the $\:0.014-10\mathrm{\:MeV}$ energy spectrum. As predicted by the Bethe–Bloch framework, the $\:\mathrm{MSP}$ profiles exhibit a classic Bragg peak at approximately 0.6–0.8 MeV, where alpha particles undergo intense Coulomb interactions with target electrons and consequently lose energy rapidly. Beyond this region, the MSP decreases with increasing particle energy because faster alpha particles spend less time interacting with the target electrons. The MSP at the Bragg peak decreases slightly from about 1.78 MeV·cm²/g for MC-W00 to 1.58 MeV·cm²/g for MC-W35. This decrease relates mainly to the lower Z/A ratio of tungsten compared with the hydrogen-rich organic matrix. In contrast, the higher density of the WO₃-rich composites strongly reduces the physical penetration depth. At 10 MeV, the projected range decreases from about 50 μm for MC-W00 to 36 μm for MC-W35. Thus, increasing WO₃ loading slightly reduces the mass stopping power but notably enhances the spatial stopping efficiency through increased material density, making MC-W35 the most effective formulation for limiting alpha-particle penetration^37,47^.

#### Proton (H^+^)

**Fig. 7:**
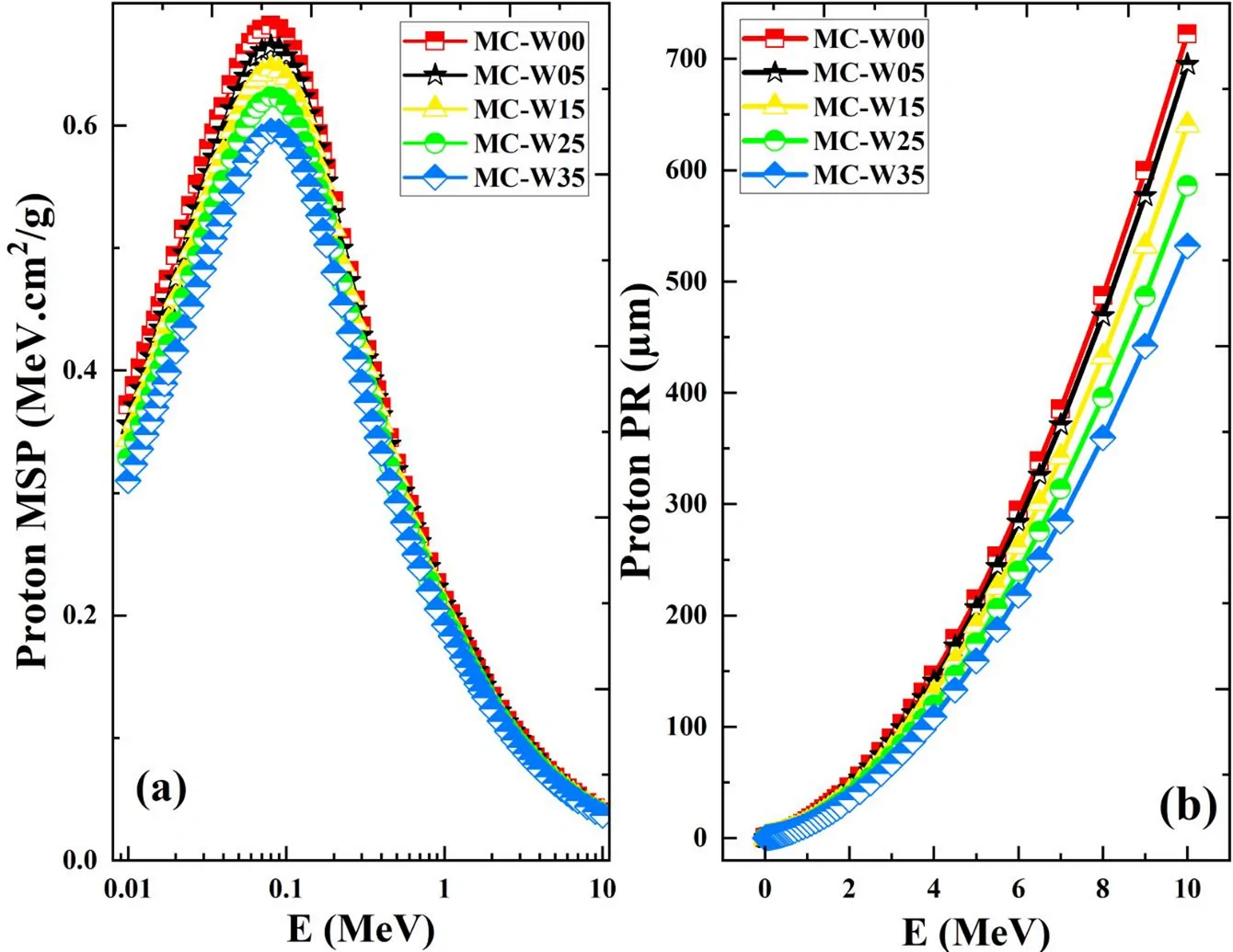
Proton (**a**) mass stopping power (MSP, MeV·cm²/g) and (**b**) projected range (PR, µm) as a function of particle energy (MeV) for MC-W00–MC-W35 samples.

Figure 7 displays the mass stopping power (MSP, MeV·cm²/g) and projected range (PR, µm) of incident protons across the investigated energy spectrum. However, their much lower mass compared with alpha particles ($\:{\mathrm{m}}_{\mathrm{p}}\approx\:{\mathrm{m}}_{{\upalpha\:}}/4$) gives protons higher velocities at comparable kinetic energies, altering their interaction dynamics and placing the Bragg peak at a significantly lower kinetic energy of about 0.08 MeV. Above the peak, the increasing proton velocity reduces the stopping power and causes a gradual decline in MSP. Increasing the WO₃ fraction slightly lowers the MSP, with the peak value decreasing from about 0.67 MeV·cm²/g for MC-W00 to 0.59 MeV·cm²/g for MC-W35. This reduction reflects the lower stopping power per unit mass of the high-Z tungsten-containing composition compared with the hydrogen-rich organic matrix.

In contrast, the projected range shows a pronounced dependence on WO₃ loading. At 10 MeV, the proton range decreases from about 720 μm for MC-W00 to 530 μm for MC-W35. The higher density of the WO₃-rich composite increases the number of target atoms and electrons encountered along the proton path, enhancing cumulative energy loss per unit distance. Therefore, although the mass stopping power changes only modestly, the increased density considerably improves the spatial stopping efficiency. MC-W35 consequently provides the highest reduction in proton penetration depth among the studied formulations, indicating its potential for use in compact radiation-shielding materials^37^.

#### Beta particles (β^-^)

**Fig. 8:**
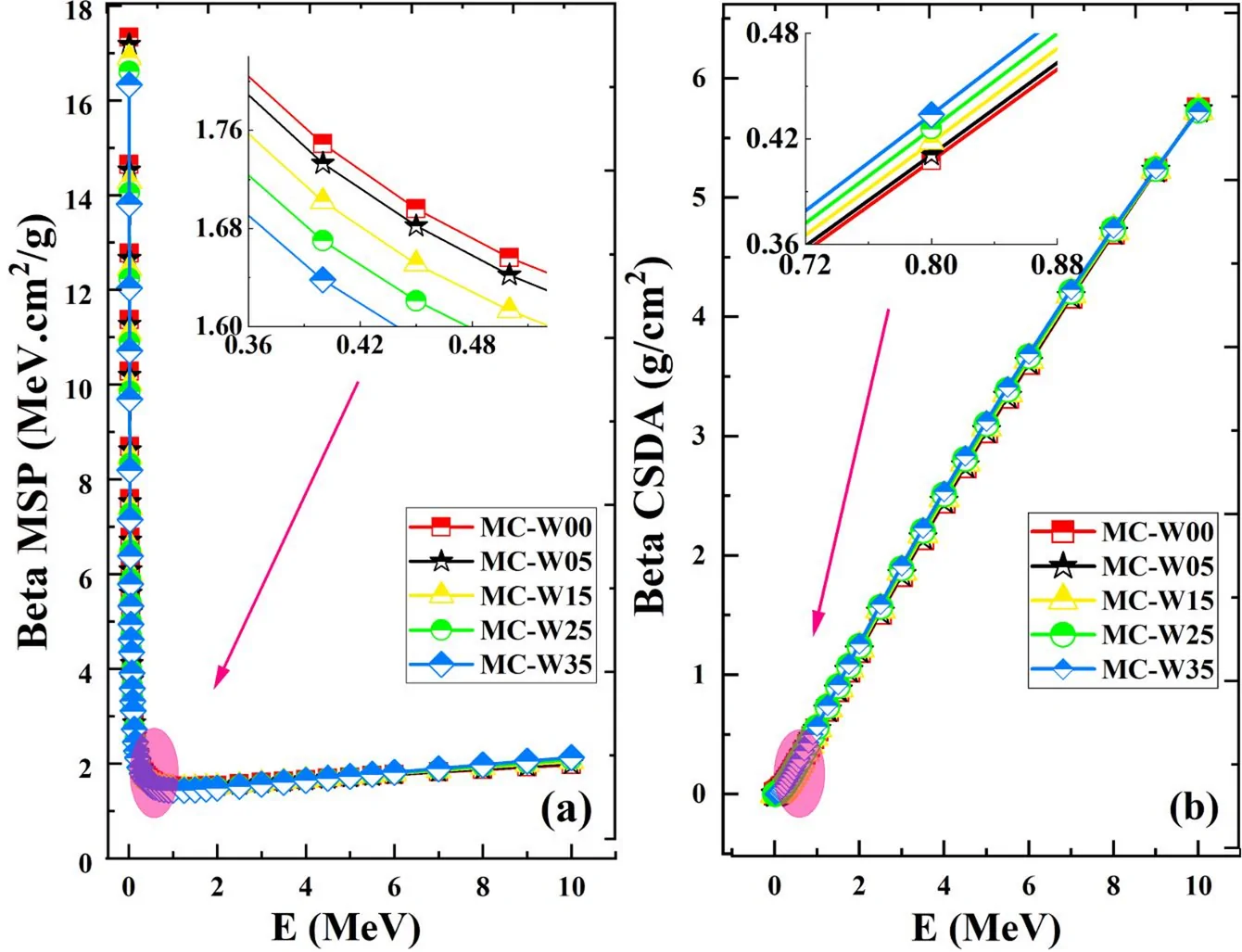
Beta (**a**) mass stopping power (MSP, MeV·cm²/g) and (**b**) continuous stopping down approximation (CSDA, g/cm²) as a function of particle energy (MeV) for MC-W00–MC-W35 samples.

Figure 8 illustrates the mass stopping power ($\:\mathrm{MSP}$, Fig. 8a) and continuous slowing down approximation range ($\:\mathrm{CSDA}$, Fig. 8b) for beta particles across the $\:0.01-10\mathrm{\:MeV}$ energy spectrum. Unlike heavy charged ions, electrons exhibit low mass ($\:{\mathrm{m}}_{\mathrm{e}}$), undergoing significant trajectory deflection during interactions. At energies below $\:1\mathrm{\:MeV}$, collision ionization losses dominate, causing a steep initial decay in $\:\mathrm{MSP}$ as velocity increases and reduces electron interaction dwell time. Within this ionization-dominated region, the insert in Fig. 8a reveals that MC-W00 yields slightly higher $\:\mathrm{MSP}$ values per unit mass compared to MC-W35 (e.g., * 1.76* vs. $\:1.60\mathrm{\:MeV}\cdot\:{\mathrm{cm}}^{2}/\mathrm{g}$ at $\:0.38\mathrm{\:MeV}$). This stems directly from the higher atomic number-to-mass ratio ($\:\mathrm{Z}/\mathrm{A}\approx\:0.50$) of the organic matrix compared to heavy tungsten ($\:\mathrm{Z}/\mathrm{A}\approx\:0.40$). Beyond $\:1\mathrm{\:MeV}$, the rate of collisional energy loss stabilizes, and radiative loss mechanisms via Bremsstrahlung emissions become increasingly active. Because radiative interactions scale with $\:{\mathrm{Z}}^{2}$, the heavy tungsten inclusions ($\:\mathrm{Z}=74$) enhance radiative attenuation at high energies, bringing the $\:\mathrm{MSP}$ curves into close convergence. In Fig. 8b, while the mass-normalized range ($\:\mathrm{CSDA}$ in $\:{\mathrm{g/cm}}^{2}$) increases linearly with energy, converting these values to physical penetration depth ($\:\mathrm{cm}$) by incorporating bulk density ($\:{\uprho\:}$) confirms that the dense MC-W35 formulation yields the shortest path length. Consequently, MC-W35 shows a shorter calculated penetration path and lower predicted secondary-radiation yield at the investigated energies (Abdelgawad et al.^39^,).

**Fig. 9:**
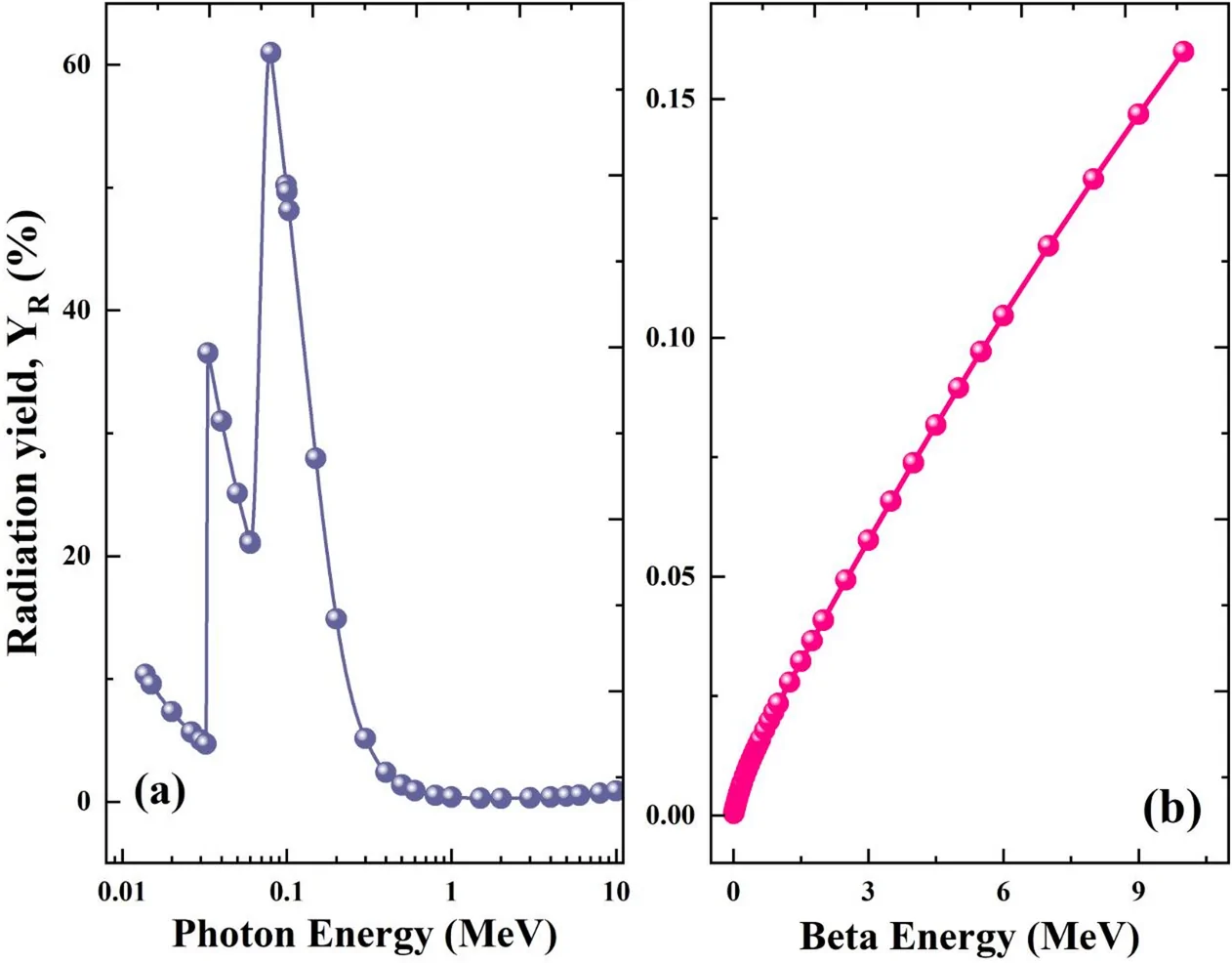
Radiation yield (Y_R_) as a function of incident particle energy for the MC-W35 composite: (**a**) photon-induced radiation obtained from MCNP and (**b**) electron-induced radiation obtained from ESTAR.

Secondary radiation generation was further examined for the MC-W35 composite using the radiation yield (Y_R_), as shown in Fig. 9. The photon-induced radiation yield shows a strong energy dependence. It reaches a maximum of about 61% near 0.08 MeV, which can be associated with enhanced photoelectric interactions and characteristic X-ray fluorescence from the high-Z constituents, particularly the tungsten edge (69.5 keV) and iodine edge (33.2 keV). At higher photon energies, it decreases rapidly and remains close to zero above 1 MeV, where Compton scattering becomes increasingly important. In contrast, the electron-induced radiation yield increases gradually with electron energy and reaches approximately 0.16% at 10 MeV. These results demonstrate that the radiation yield depends strongly on the incident particle type and energy. The low electron-induced yield indicates limited radiative energy loss within the studied energy range^48–52^.

### Practical feasibility, limitations, and future outlook

Although the present work is entirely computational, the proposed DDQ/LiI/$\:{\mathrm{WO}}_{3}$ composite system is considered practically feasible for experimental fabrication, as its constituents are readily available solid precursors that can be processed using standard composite processing techniques. The mass fractions and overall slab thickness can be systematically tailored to meet specific shielding demands.

Nevertheless, several limitations inherent to theoretical modeling should be acknowledged. The simulations assume ideally homogeneous and defect-free material matrices. In practice, real composite formulations may exhibit particle agglomeration, interfacial boundary effects, or localized composition variations that could subtly alter radiation transport. Furthermore, the present study does not evaluate experimental microstructural homogeneity, nor does it assess long-term mechanical, thermal, electrical, or radiation-aging stability under prolonged exposure.

Consequently, future investigation should prioritize experimental synthesis, microstructural characterization, and irradiation testing under realistic mixed-radiation conditions. Coupling these experimental steps with device-level evaluations will be critical to verifying whether the predicted photon shielding efficacy translates into promising encapsulation systems for practical electronic protection.

## Conclusions

This study established DDQ/LiI/WO₃ hybrid composites as a promising multifunctional platform for radiation shielding of electronic systems operating in radiation-harsh environments. By integrating a charge-transfer organic matrix (DDQ) with high-Z tungsten trioxide (WO₃) fillers and lithium iodide (LiI), the proposed hybrid architecture demonstrates effective shielding capability against photons, fast neutrons, protons, alpha particles, and beta particles within a single material system. Systematic optimization demonstrated that increasing WO₃ loading consistently enhances photon attenuation while maintaining a low material density and favorable mass efficiency. Among the investigated compositions, MC-W35 provided the most favorable overall shielding performance, combining high photon attenuation with a low areal density per half-value layer. The significance of these findings extends beyond the improvement of individual shielding parameters. The complementary radiation-interaction characteristics of DDQ, LiI, and WO₃ enable different shielding functions to be integrated within the same architecture, reducing the need to treat radiation protection as a separate external barrier. In this context, the proposed system provides a potential route toward intrinsic self-shielding and radiation-protective encapsulation, where the material surrounding an electronic component contributes directly to its radiation protection while retaining the functional characteristics required for electronic applications. The low average atomic number of the organic matrix also limits radiative energy-loss mechanisms associated with charged-particle interactions, supporting the low secondary-radiation yield predicted for the composite. Overall, DDQ/LiI/WO₃ composites offer a favorable balance between radiation attenuation and radiotherapy mass efficiency, establishing a solid physics-based framework for electronic systems exposed to radiation in space, nuclear, radiotherapy, and industrial environments.

## Supplementary Information

Below is the link to the electronic supplementary material.

Supplementary Material 1

Supplementary Material 2

## Data Availability

The datasets generated and/or analyzed during the current study are available from the corresponding author on reasonable request.
