# Supplementary material for: Computational study of hybrid DDQ/LiI/WO₃ composites for multi-radiation shielding in microelectronic applications
Source: Sci Rep. 2026 Sep 8;16:28107. doi: 10.1038/s41598-026-68436-3 (PMC13554150; doi:10.1038/s41598-026-68436-3)
Supplement: Supplementary file 1 [file 41598_2026_68436_MOESM1_ESM.docx]

**Reviewer 1**

**We sincerely thank the reviewer for the careful and constructive evaluation of our manuscript. We greatly appreciate the valuable comments and suggestions, which have helped us improve the manuscript. We have carefully addressed all the raised points, and our detailed responses are provided below and all corresponding changes have been highlighted in the revised manuscript in yellow.**

1.Please shorten the title.

**The authors:**

We appreciate the reviewer's helpful suggestion. The title has been revised to make it shorter, clearer, and more concise while preserving the scientific scope of the study. In addition, the title has been reformulated as a single statement, consistent with the journal's editorial recommendation.

2.In the last paragraph of introduction, please clarify the research gap.

**The authors:**

We thank the reviewer for this insightful comment. The Introduction has been substantially revised and reorganized in response to the reviewers’ comments to improve its coherence, scientific context, and logical flow. In the revised version, the research gap is no longer presented only in the final paragraph; instead, it is introduced and developed in the third paragraph, where the limitations of conventional shielding approaches and the need for multifunctional materials for radiation-tolerant electronic systems are clearly highlighted. The objective and scientific contribution of the present study are then presented accordingly in the following paragraph

**The corresponding changes have been highlighted in the revised manuscript (introduction- Page 3, line 8)**

3.Standardize abbreviations.

**The authors:**

We appreciate the reviewer's careful observation. The manuscript has been thoroughly revised to ensure the consistent use of abbreviations throughout the text. All abbreviations are now defined at their first occurrence and used consistently thereafter.

4.Clearly justify the novelty of DDQ/LiI/WO₃ over existing shielding systems.

**The authors:**

We thank the reviewer for this valuable comment. The last paragraph of the Introduction has been revised to better justify the novelty of the proposed DDQ/LiI/WO₃ hybrid composite. The revised text now clarifies the scientific rationale behind integrating these constituents within a single hybrid architecture and highlights how this design differs from conventional shielding systems. **The corresponding changes have been highlighted in the revised manuscript (introduction- Page 3, line 17)**

5.Replace "developed" with "proposed" throughout.

**The authors:**

We thank the reviewer for this helpful suggestion. The manuscript has been carefully revised, and terminology implying experimental fabrication has been replaced with wording appropriate for a simulation-based study. In particular, *developed* has been replaced with *proposed*, where appropriate, throughout the manuscript.

6.Expand discussion of space radiation environment.

**The authors:**

We thank the reviewer for this comment. In accordance with the major revisions requested by Reviewer 2-comment 11, the primary title, objective, and scope of this manuscript have been fundamentally reframed away from exclusive deep-space environment applications.

Rather than restricting the manuscript to high-energy cosmic rays, the focus has been expanded to evaluate the $0.01-10\text{ MeV}$ shielding performance for sensitive microelectronic components across a wider range of radiation-rich environments—including radiation therapy facilities, nuclear reactor instrumentation, aviation, and general electronic protection.

Consequently, references exclusively detailing the deep-space radiation environment have been replaced with a broader discussion on multi-environment electronic protection against photon and ionizing radiation.

7.Add uncertainty and sensitivity analyses.

**The authors:**

We thank the reviewer for this valuable guidance on improving statistical robustness and compositional analysis. We have addressed both aspects in the revised manuscript as follows:

1. Uncertainty Analysis:

Following a complete re-evaluation of the MCNP5 output files ($5\times{10}^{6}$ particle histories), we have updated the uncertainty section to reflect the energy-dependent relative errors (FSD). For the primary spectrum ($0.1-10\text{ MeV}$), the statistical error was exceptionally low ($\text{FSD}<0.6\%$). At lower energies ($0.04-0.1\text{ MeV}$), higher statistical uncertainties occurred (peaking at $12.6\%$). All corresponding text sections have been revised accordingly.

2. Sensitivity Analysis:

A dedicated compositional sensitivity analysis has been incorporated and illustrated in a new figure (Figure 5). This analysis quantitatively evaluates how variations in filler loading impact the key attenuation parameters.

**The corresponding changes have been highlighted in the revised manuscript (Material and methods-Page 6, line 24--Results- Page 9, line 28-page 12, line 12)**

8.Provide detailed MCNP input parameters.

**The authors:**

we appreciate the reviewer’s constructive suggestion. The MCNP methodology has been revised to provide a more detailed description of the simulation setup. Additional information regarding the source configuration, simulation geometry, photon transport mode, tally specification (F1:P), particle importance variance-reduction technique (IMP:P), number of particle histories, and statistical uncertainty has been included to improve the clarity and reproducibility of the computational procedure. **The corresponding changes have been highlighted in the revised manuscript (Material and methods-Page 6, line 10)**

9.In introduction, state and mention, shortly or deeply, the advantages and disadvantages of radiation shielding for different materials such as:
•Glasses and alloys (use the following references):
https://doi.org/10.1016/j.radphyschem.2026.113950.
https://doi.org/10.1016/j.net.2026.104180.
https://doi.org/10.1038/s41598-025-33307-w
https://doi.org/10.1016/j.radphyschem.2025.113396
https://doi.org/10.1016/j.radphyschem.2025.113216
•Alloys (use the following references):
10.3934/matersci.2025053
https://doi.org/10.1016/j.radphyschem.2025.113405.
https://doi.org/10.1016/j.apradiso.2025.112353.
•Nanocomposites (use the following reference):
https://doi.org/10.1016/j.radphyschem.2025.113078
•Ferrites (use the following reference):
https://doi.org/10.1364/OME.521679
•Single crystals (use the following references):
http://dx.doi.org/10.1016/j.anucene.2013.04.024
<http://dx.doi.org/10.1016/j.anucene.2011.08.001>

**The authors:**

We sincerely thank the reviewer for this valuable suggestion. The Introduction has been substantially revised and reorganized to address this comment and the related recommendations of the reviewers. The revised Introduction now includes a concise discussion of the advantages and limitations of the main conventional radiation-shielding materials, including glasses, metallic alloys, nanocomposites, ferrites, and single crystals. The references suggested by the reviewer have also been incorporated at the relevant points to support this discussion. These revisions provide a broader and more up-to-date context for the development of the proposed hybrid shielding system. **The corresponding changes have been highlighted in the revised manuscript (introduction- Page 2, line 15)**

10.Include comparison plots between MCNP and PHY-X.

**The authors:**

We would like to clarify that the direct comparison between Monte Carlo simulation outputs and Phy-X/PSD theoretical database predictions is already established in **Figure 1** of the manuscript.

11.Discuss limitations of homogeneous material assumptions.

**The authors:**

We thank the reviewer for highlighting this critical physical constraint. The limitations of the homogeneous-material assumption have been added to the new “Limitations and Future Outlook” section, including the possible effects of particle agglomeration, interfacial effects, and local compositional variations on radiation transport. **The corresponding changes have been highlighted in the revised manuscript (Results- Page 15, line 10)**

12.In introduction, state and mention, shortly or deeply about different methods used in radiation shielding such as:
Experimental method (use the following references):
http://dx.doi.org/10.21203/rs.3.rs-3925330/v1
https://doi.org/10.1016/j.radphyschem.2022.110586
https://doi.org/10.1016/j.net.2024.03.038
https://doi.org/10.1007/s00339-023-06681-3
•Theoretical method (use the following references):
https://doi.org/10.1007/s10854-022-09604-9
https://doi.org/10.1016/j.radphyschem.2025.112510
https://doi.org/10.1016/j.radphyschem.2024.111726
•Simulation method (use the following references):
https://doi.org/10.21608/ajnsa.2021.80370.1478
https://doi.org/10.1016/j.pnucene.2022.104482
<https://doi.org/10.1016/j.radphyschem.2024.112155>

**The authors:**

We sincerely thank the reviewer for this valuable suggestion. The Introduction has been substantially revised and reorganized to address this comment. A concise discussion of the main approaches used to investigate radiation-shielding materials has been added, covering experimental measurements, theoretical calculations, and radiation-transport simulations. The relevant references suggested by the reviewer have also been incorporated to support these approaches. This revision clarifies the methodological framework commonly used for evaluating radiation-shielding performance and provides a clearer context for the computational methodology adopted in the present study. **The corresponding changes have been highlighted in the revised manuscript (introduction- Page 3, line 28)**

13.Moderate all overly strong claims.

**The authors:**

We thank the reviewer for this valuable advice on scientific presentation. The manuscript has been carefully revised to tone down definitive or overly absolute assertions throughout the all sections.

14.Expand comparison with recent shielding materials.

**The authors:**

We appreciate the reviewer’s valuable suggestion. The comparison with reference shielding materials has been expanded in the revised manuscript and table (2) by adding ordinary concrete and lead to the materials already considered, including epoxy resin, polyimide (Kapton), silicon, and tantalum. These materials were selected to provide a broader comparison with established shielding materials and materials commonly used in electronics applications. The comparison was performed at the same photon energy (0.1 MeV), and the corresponding discussion has been expanded to provide a more comprehensive assessment of the attenuation performance and density of the proposed MC-W35 composite. **The corresponding changes have been highlighted in the revised manuscript (Results- Page 10, line 16)**

15.Discuss secondary radiation generation.

**The authors:**

We sincerely thank the reviewer for raising this critical point regarding radiation transport physics and material safety design. We have thoroughly addressed secondary radiation generation by incorporating a quantitative analysis of the radiation yield ($Y_{R}$) for both incident photons and electrons across $0.01381\text{--}10\text{ MeV}$, alongside adding **Fig. 9** and dedicated text in the revised manuscript. **The corresponding changes have been highlighted in the revised manuscript (Results- Page 14, line 22)**

16.Explain practical fabrication feasibility.

**The authors:**

We thank the reviewer for this important remark regarding practical implementation. The practical fabrication feasibility of the proposed DDQ/LiI/WO₃ composite has now been discussed in the revised manuscript. Since the present study is entirely simulation-based, experimental fabrication was not performed. The revised discussion explains the feasibility of formulating the solid constituents with controlled compositions and adjustable material thickness, while experimental fabrication and characterization are identified as important steps for future validation of the predicted shielding performance. **The corresponding changes have been highlighted in the revised manuscript (Results- Page 15, line 5)**

17.According to introduction section, you should discuss thermal and mechanical properties.

**The authors:**

We sincerely thank the reviewer for this valuable suggestion. The Introduction has been substantially revised to emphasize that the suitability of a material for self-shielding or encapsulation of electronic systems cannot be judged solely by its radiation-attenuation capability. The revised text now highlights the importance of adequate mechanical integrity and thermal stability, together with electrical compatibility and structural homogeneity, for maintaining reliable protection and device performance under radiation-harsh operating conditions. These considerations have also been incorporated into the discussion of the research gap and the rationale for developing the proposed DDQ/LiI/WO₃ hybrid composite. **The corresponding changes have been highlighted in the revised manuscript (introduction- Page 3, line 9)**

18.Include areal density comparison.

**The authors:**

We appreciate the reviewer’s suggestion. The areal density corresponding to one half-value layer (HVL) has now been included in **Table 2** to provide a more meaningful assessment of shielding mass efficiency. The areal density was calculated, and the corresponding discussion has been added to the revised manuscript. This comparison shows that MC-W35 requires substantially lower areal density per HVL than the conventional polymeric and concrete materials considered, highlighting its potential for weight-conscious radiation shielding applications. **The corresponding changes have been highlighted in the revised manuscript (Results- Page 10, line 16)**

19.Improve physical interpretation of all shielding mechanisms.

**The authors:**

We thank the reviewer for this insightful comment. The manuscript has been thoroughly revised, and the discussions for all relevant figures (including photon, neutron, alpha, proton, and beta interaction profiles) have been carefully updated.

20.Improve figure quality.

**The authors:**

We thank the reviewer for this helpful suggestion. The quality and resolution of all figures have been carefully improved in the revised manuscript to ensure clearer presentation of the results and better readability of the graphical elements.

21.Correct typographical errors.

**The authors:**

We thank the reviewer for drawing our attention to the importance of careful presentation. The manuscript has been thoroughly proofread, and the identified typographical, spelling, and formatting errors have been corrected throughout the revised version.

22.Update recent references.

**The authors:**

We are grateful to the reviewer for this valuable recommendation. Several older references have been replaced with more recent and relevant studies, and a number of the references suggested by the reviewer have also been incorporated. As a result, the reference list has been substantially updated and improved to provide a more current and comprehensive representation of the relevant literature.

23.Improve consistency of notation.

**The authors:**

We thank the reviewer for this helpful observation. The manuscript has been carefully checked to ensure consistent notation throughout the text, equations, tables, and figures. Symbols, abbreviations, subscripts, and units have been standardized wherever necessary to improve clarity and consistency.

24.Add limitations section.

**The authors:**

We sincerely thank the reviewer for this valuable comment. A dedicated “**Practical Feasibility, Limitations, and Future Outlook**” section has been added to the revised manuscript. The new section explicitly acknowledges the computational nature of the present study and discusses the limitations associated with the assumptions of homogeneous composition, as well as the need to experimentally verify structural homogeneity, mechanical, thermal, electrical, and radiation-aging stability. It also outlines the main directions for future experimental validation under realistic radiation conditions and device-level assessment of the proposed composites. **The corresponding changes have been highlighted in the revised manuscript (Results- Page 15, line 4)**

25.Improve conclusion wording.

**The authors:**

We express our sincere gratitude to the reviewer for this constructive feedback. The **Conclusions** section (Section 4) has been completely rewritten to eliminate repetitive numerical lists and present a synthesized, high-impact assessment of the study's core contributions and physical mechanisms.
