# Supplementary material for: Computational study of hybrid DDQ/LiI/WO₃ composites for multi-radiation shielding in microelectronic applications
Source: Sci Rep. 2026 Sep 8;16:28107. doi: 10.1038/s41598-026-68436-3 (PMC13554150; doi:10.1038/s41598-026-68436-3)
Supplement: Supplementary file 2 [file 41598_2026_68436_MOESM2_ESM.docx]

**Reviewer 2**

**We sincerely thank the reviewer for the thorough and constructive evaluation of our manuscript. The insightful comments and suggestions have been extremely helpful in improving the scientific quality, clarity, and reproducibility of the work. All comments have been carefully addressed, and the manuscript has been revised accordingly. A detailed point-by-point response is provided below, and all modifications have been highlighted in the revised manuscript in turquoise.**

1- The title needs to be revised to accurately reflect the scope and approach of the study. Currently, the phrase “Organo-Inorganic Synthesis” is used and then the phrase “(DDQ/LiI/WO₃) Systems” is repeated repeatedly, which causes confusion and reduces the clarity of the title. Choose a single, coherent framework—either a broader synthetic concept or a specific chemical system—and rewrite the title accordingly.

**The authors:**

We appreciate the reviewer's helpful suggestion. The title has been revised to make it shorter, clearer, and more concise while preserving the scientific scope of the study. In addition, the title has been reformulated as a single statement, consistent with the journal's editorial recommendation.

2- The abstract does not currently reflect the logical structure of the paper. It should explicitly present both quantitative and qualitative results and offer a synthesized interpretation of the findings, rather than merely describing the outcomes without contextual meaning.

**The authors:**

We sincerely thank the reviewer for this constructive suggestion regarding the structural logic and contextual impact of the Abstract. We have thoroughly rewritten the Abstract to reflect a cohesive narrative, integrating both quantitative metrics and qualitative physical interpretations.

Furthermore, in alignment with the **Comment 11**, we have broadened the scope and primary rationale of the study. The applicability of the proposed DDQ/LiI/$\text{WO}_{3}$ hybrid composite is no longer restricted solely to space missions but now encompasses sensitive electronic devices operating across diverse radiation-harsh environments, including radiotherapy facilities, nuclear reactors, and aerospace applications.

3- The introduction lacks coherence, clarity, and proper reasoning. Specifically, it does not adequately discuss the effects of various materials on radiation attenuation. Moreover, the descriptions of isothermal coatings and polymeric composite materials are incomplete, and their applications in aircraft, self-shielding, and fabricated parts are mentioned without sufficient explanation or context. In addition, several references are outdated or irrelevant and should be removed, while others are incomplete and require revision. I encourage the authors to replace these with more current and pertinent sources. To assist in strengthening this section, I have provided a list of suggested references below.
• https://doi.org/10.1038/s41598-025-94120-z
• https://doi.org/10.1007/s40042-025-01405-7
• https://doi.org/10.1016/j.cej.2026.176910
• https://doi.org/10.1016/j.radphyschem.2025.113023
• https://doi.org/10.1016/j.nimb.2025.165921
• https://doi.org/10.1140/epjp/s13360-025-06700-7
• <https://doi.org/10.1016/j.asr.2025.06.078>

**The authors:**

We sincerely thank the reviewer for this valuable and constructive comment. The Introduction has been completely rewritten and substantially reorganized to improve its coherence, clarity, scientific reasoning, and logical flow. The revised version now provides a clearer discussion of radiation-shielding materials, self-shielding concepts, material requirements, and the advantages and limitations of different shielding approaches. The outdated or less relevant references have also been replaced where appropriate, and the recent references suggested by the reviewer have been incorporated into the revised Introduction.

4- The purpose of the study and the novelty of the work are not clearly articulated in the current manuscript. I recommend that the authors explicitly state the research objectives and clearly distinguish their contribution from previous work. This is particularly important in the introduction, where the gap in the literature should be identified and the specific aims of the study should be presented in a concise and unambiguous manner.

**The authors:**

We sincerely thank the reviewer for this crucial recommendation. The closing sections of the Introduction (Section 1) have been thoroughly restructured to explicitly define the research gap, articulate the unique novelty, and present the specific objectives of this study in a concise, unambiguous manner. **The corresponding changes have been highlighted in the revised manuscript (introduction- Page 3, line 17)**

5- The section on combinatorial modeling and sample design lacks sufficient detail on density calculations. Authors should address any ambiguities in the text to improve reproducibility.

**The authors:**

We thank the reviewer for pointing out the need for clearer mathematical detail regarding density calculation. In the revised manuscript, we have explicitly provided the complete formulation of the inverse rule of mixtures used to determine the theoretical composite densities ($\rho_{\text{theo}}$). Additionally, we have explicitly specified the reference theoretical density values applied for each raw constituent component eliminating any ambiguity and ensuring full reproducibility of the reported sample properties in Table 1. **The corresponding changes have been highlighted in the revised manuscript (Materials & Methods- Page 5, line 10)**

6- To enhance the overall quality and readability of the manuscript, I suggest that the authors carefully proofread the text to correct grammatical and spelling errors. I also recommend converting any Arabic numerals to standard numerals for consistency with international scientific standards.

**The authors:**

We thank the reviewer for drawing our attention to the importance of careful presentation. The manuscript has been thoroughly proofread, and the identified typographical, spelling, and formatting errors have been corrected throughout the revised version.

7- The sections on shielding quantities, equations, and simulation currently lack coherence and logical flow. I recommend reorganizing this part of the manuscript into two distinct, clearly defined sections: one dedicated to the simulation setup and methodology, and another focused on shielding quantities and the associated equations. Additionally, the equations section should briefly address attenuation analysis to ensure completeness and proper contextualization. This restructuring would resolve the current inconsistency and improve the overall readability of the methodology.

**The authors:**

We sincerely thank the reviewer for this constructive structural recommendation. We have completely reorganized the methodology section to enhance logical flow and clarity as requested:

1. Section 2.3.1 (Monte Carlo Simulation Setup)
2. Section 2.3.2 (Radiation Shielding Quantities and Attenuation Analysis)

This restructuring successfully removes previous inconsistencies and significantly improves the readability of our methodology.

**The corresponding changes have been highlighted in the revised manuscript (Materials & Methods- Page 6)**

8- The manuscript contains several instances where terms such as "measurement methodology" and related synonyms are used to describe the work. However, this study is based entirely on simulations, and no experimental measurements were performed. This inconsistency creates ambiguity and could mislead readers regarding the nature of the study. I strongly recommend that the authors carefully revise the text to replace all such terminology with appropriate simulation-based language (e.g., "simulation methodology," "computational approach," or "modeling framework") throughout the manuscript.

**The authors:**

We thank the reviewer for pointing out this critical terminology issue. We fully agree that using measurement-related terms in a purely simulation-based study creates unnecessary ambiguity. The manuscript has been thoroughly reviewed and revised to ensure consistent, strictly computational language throughout.

9- The section entitled "Density and compositional effects" should be relocated to the Methods section. Its current placement disrupts the logical flow of the manuscript, as it describes methodological considerations rather than presenting simulation results.

**The authors:**

We thank the reviewer for this constructive suggestion regarding the manuscript structure. As requested, the section "Density and compositional effects" has been relocated to the Materials and Methods section (Subsection 2.2). This adjustment successfully eliminates the disruption in logical flow and ensures that all methodological and compositional design considerations are presented together prior to the simulation results.**The corresponding changes have been highlighted in the revised manuscript (Materials & Methods- Page 5, line 19)**

10- The explanation of Figure 1 is inconsistent with the presented results, which introduces significant ambiguity. Specifically, the authors state that the statistical uncertainty—expressed as fractional standard deviation (FSD)—is consistently below 2% (i.e., FSD < 0.02) throughout the study. However, the figure itself does not display or support this claim. Additionally, if this level of precision is a key feature of the results, it should be clearly presented or referenced within the figure itself (e.g., as error bars, a dedicated panel, or a supplementary table).

**The authors:**

We express our sincere gratitude to the reviewer for this crucial observation. Following this comment, we performed a thorough re-examination of all raw MCNP output files across the evaluated energy spectrum. The reviewer was entirely correct: the blanket statement claiming an $\text{FSD}<2\%$ across the entire spectrum was inaccurate.

Our re-evaluation confirmed that:

1. At lower diagnostic energies ($0.04-0.1\text{ MeV}$), statistical uncertainties exceed $2\%$ (peaking at $12.6\%$) due to intense photoelectric absorption, which drastically suppresses transmitted photon current (low tally scoring histories) through high-$Z$ composite compositions.
2. For the main spectrum ($0.1-10\text{ MeV}$), the statistical error is indeed exceptionally low, remaining strictly below $0.6\%$ ($\text{FSD}<0.006$) and reaching near-zero values at higher kinetic energies.

To resolve all ambiguities and ensure full consistency between the text and output data, we have executed two specific manuscript revisions and added a supplementary table:

- Revision 1 (Section B: Simulation Parameters)
- Revision 2 (Figure 1 Discussion)
- Supplementary Data (Table S1):

| **Energy (MeV)** | **S1 (%)** | **S2 (%)** | **S3 (%)** | **S4 (%)** | **S5 (%)** |
| --- | --- | --- | --- | --- | --- |
| 0.0332 | 12.6 | 12 | 50 | 10.72 | 12 |
| 0.04 | 2.06 | 3.98 | 12.62 | 11.72 | 10.74 |
| 0.05 | 0.4 | 0.57 | 1.31 | 3.61 | 12.6 |
| 0.05954 | 0.18 | 0.23 | 0.39 | 0.74 | 1.66 |
| 0.06 | 0.17 | 0.22 | 0.37 | 0.7 | 1.54 |
| 0.08 | 0.08 | 0.13 | 0.33 | 0.99 | 3.9 |
| 0.09897 | 0.05 | 0.08 | 0.14 | 0.28 | 0.62 |
| 0.1 | **0.05** | **0.07** | **0.14** | **0.26** | **0.58** |
| 0.103 | 0.05 | 0.07 | 0.13 | 0.23 | 0.48 |
| 0.15 | 0.03 | 0.04 | 0.06 | 0.08 | 0.11 |
| 0.2 | 0.03 | 0.03 | 0.04 | 0.05 | 0.06 |
| 0.3 | 0.02 | 0.02 | 0.03 | 0.03 | 0.04 |
| 0.4 | 0.02 | 0.02 | 0.02 | 0.03 | 0.03 |
| 0.5 | 0.02 | 0.02 | 0.02 | 0.02 | 0.03 |
| 0.6 | 0.02 | 0.02 | 0.02 | 0.02 | 0.02 |
| 0.8 | 0.02 | 0.02 | 0.02 | 0.02 | 0.02 |
| 1 | 0.02 | 0.02 | 0.02 | 0.02 | 0.02 |
| 1.5 | 0.01 | 0.01 | 0.02 | 0.02 | 0.02 |
| 2 | 0.01 | 0.01 | 0.01 | 0.02 | 0.02 |
| 3 | 0.01 | 0.01 | 0.01 | 0.01 | 0.02 |
| 4 | 0.01 | 0.01 | 0.01 | 0.01 | 0.01 |
| 5 | 0.01 | 0.01 | 0.01 | 0.01 | 0.01 |
| 6 | 0.01 | 0.01 | 0.01 | 0.01 | 0.01 |
| 8 | 0 | 0.01 | 0.01 | 0.01 | 0.01 |
| 10 | 0 | 0.01 | 0.01 | 0.01 | 0.01 |

**The corresponding changes have been highlighted in the revised manuscript (Results- Page 9, line 28-page 12, line 12)**

11- A more fundamental concern relates to the scope of the study relative to its stated title and aims. The title explicitly references "Space Electronic Applications," yet the results presented in Figures 2 through 7 focus exclusively on photon energies up to 10 MeV, with no consideration of space-relevant energy. This energy range and simulation approach are more characteristic of medical or terrestrial radiation shielding applications than of the space radiation environment, which encompasses a much broader spectrum of particles and energies. The authors should either:
Expand their study to include space-relevant particles and higher energy ranges, or
Revise the title and framing of the article to accurately reflect the actual scope of the work (e.g., focusing on photon shielding for medical or general radiation protection applications).
Without such revision, the current title is misleading and does not align with the presented content.

**The authors:**

we are deeply grateful to the reviewer for drawing our attention to this pivotal point. We fully agree that restricting the manuscript's framing strictly to space applications was unnecessarily limiting and did not accurately reflect the broader applicability of our $0.01-10\text{ MeV}$ photon shielding results.

In response to the reviewer’s recommendation, we have comprehensively revised the title, objectives, and framing of the manuscript to adopt a much broader and more generalized scope. Rather than restricting the application solely to space environments, the study has been refocused on the radiation protection of electronic devices and sensitive equipment operating across various radiation environments—including radiation therapy facilities, nuclear reactor instrumentation, and general electronic shielding.

12- The conclusion section currently lacks a clear approach and largely reiterates content already presented in the results and discussion sections. Rather than simply restating earlier findings, the conclusion should concisely synthesize the key points of the study, highlight the main contributions, and provide an overall final assessment of the work. I recommend that the authors include brief remarks on the significance of their findings, their implications for the field, and potential directions for future research. This would give the section a more impactful and conclusive character.

**The authors:**

We express our sincere gratitude to the reviewer for this constructive feedback. The **Conclusions** section (Section 4) has been completely rewritten to eliminate repetitive numerical lists and present a synthesized, high-impact assessment of the study's core contributions and physical mechanisms.
